# Enhanced Optoelectronic Response of TiO_2_ Photodetector Sensitized via CuInSe_2_ Quantum Dots

**DOI:** 10.3390/nano15070522

**Published:** 2025-03-30

**Authors:** Yanxu Zhang, Kexin Yu, Jin Zhao, Shuaiqi Xu, Mengqi Lv, Qiuling Zhao, Xue Du, Maorong Wang, Xia Wang

**Affiliations:** 1 Shandong Engineering Research Center of New Optoelectronic Information Technology and Devices, School of Mathematics and Physics, Qingdao University of Science & Technology, Qingdao 266061, China; 2 School of Physics and Technology, University of Jinan, Jinan 250022, China

**Keywords:** CuInSe_2_, quantum dots, TiO_2_, photodetector

## Abstract

Colloidal copper-based chalcogenide quantum dots (QDs), particularly lead-free CuInSe_2_ systems, have emerged as promising photosensitizers for optoelectronic de-vices due to their high extinction coefficients and solution processability. In this work, we demonstrate a TiO_2_ photodetector enhanced through interfacial engineering with the size of 9.88 ± 2.49 nm CuInSe_2_ QD_s_, synthesized via controlled thermal injection. The optimized device architecture combines a 160 nm TiO_2_ active layer with 60 μm horizontal channel electrodes, achieving high performance metrics. The QD-sensitized device demonstrates an impressive switching ratio of approximately 10^5^ in the 405 nm wavelength, a significant 34-times increase in responsivity at a 2 V bias, and a detection rate of 4.17 × 10^8^ Jones. Due to the limitations imposed by the TiO_2_ bandgap, the TiO_2_ photodetector exhibits a negligible increase in photocurrent at 565 nm. The engineered type-II heterostructure enables responsivity enhancement across an extended spectral range through sensitization while maintaining equivalent performance characteristics at both 405 nm and 565 nm wavelengths. Furthermore, the sensitized architecture demonstrates superior response kinetics, enhanced specific detectivity, and exceptional operational stability, establishing a universal design framework for broadband photodetection systems.

## 1. Introduction

With the rapid advancement of information technology and the growing demand for high-speed communication, optical sensors, and imaging technologies, photodetectors have become essential components in modern technological applications [1,2]. Conventional photodetectors, particularly those based on semiconductor materials like titanium dioxide (TiO_2_) [3], are extensively utilized across various fields due to their low cost, stability, and excellent chemical properties [4,5,6,7]. However, the application of TiO_2_ in photodetectors still faces some challenges, especially in terms of photoelectric conversion efficiency and optical response range. The large bandgap of TiO_2_ leads to its low absorption efficiency in the visible range, limiting its performance in low-light detection and multi-band response. To overcome this problem, Chen et al. prepared photoelectrochemical-based photodetectors based on Cu_2_O/TiO_2_ heterostructures with a special interfacial structure that accelerates the separation and transfer of photogenerated carriers [8]. Yang et al. prepared photodetectors based on TiO_2_/Bi_2_Se_3_ heterostructures, which fully utilize the light-absorbing properties of TiO_2_ to absorb more photons and generate larger photocurrents [9]. Whereas the approach of simply constructing a heterojunction has enhanced photocurrent, responsivity, and detection rate [10], there will still be a limitation on the photoresponse detection range. The work reported by Govind Gupta et al. provides an up-to-date perspective on how TiO_2_ can be structurally and electronically modified for improved device applications. By integrating these insights, our study further explores the synergistic effects of quantum dot sensitization and TiO_2_ modification, providing a new avenue for optimizing photodetector performance. Based on the unique photovoltaic properties of quantum dots (QDs) [11] high extinction coefficient, tunable bandgap, and excellent photovoltaic conversion efficiency, photons can be effectively captured and more electron-hole pairs can be generated, which can improve the responsivity and detection rate of photodetectors. Rajeev Ray et al. constructed an improved photodetector using chalcogenide QD_s_ and atomic layer deposition (ALD) ultrathin TiO_2_ films to enhance the overall performance of heterostructures photodetectors through interface engineering [12]. Qu et al. investigated Ag NPs and MoS_2_ QD_s_ dual-modified graphene/gallium arsenide near-infrared photodetectors, utilizing the dielectric confinement effect of MoS_2_ QD_s_ to enhance the performance of the photodetectors [13]. The unique quantum effect of QD_s_ makes them another effective strategy to broaden the detectable band [14], and lead-free colloidal copper-based sulfur-based QD_s_ (e.g., CuInSe_2_ QD_s_) have been widely used in optoelectronics by virtue of their low cost, lower risk of environmental contamination, and tunable bandgap [15,16]. The bandgap of CuInSe_2_ QD_s_ is highly tunable, which enables them to perform effective photovoltaic responses in different spectral ranges, especially for the absorption of near-infrared (NIR) light [17,18]. In addition, more mature synthesis methods for CuInSe_2_ QD_s_, such as thermal injection, are capable of synthesizing QD_s_ with excellent optoelectronic properties, whose size and morphology can be precisely adjusted by controlling the synthesis conditions. These excellent photovoltaic properties make CuInSe_2_ QD_s_ ideal photodetector materials [16,19,20,21], which can significantly enhance the performance of conventional photovoltaic materials such as TiO_2_.

In this study, CuInSe_2_ QD_s_ with an average size of ~9.88 nm were synthesized by thermal injection and applied to sensitization optimizing of TiO_2_ photodetectors. At 405 nm, the responsivity (*R*) and detectivity (D*) reached 4.44 × 10^−5^ mA/W and 4.17 × 10^8^ Jones. Moreover, the type-II heterostructure, which extends the detectable range to 565 nm, enables the sensitized photodetectors to exhibit significant improvements in responsivity and detectivity, highlighting the great potential of CuInSe_2_ QDs as a sensitizing material for TiO_2_ photodetectors. This work is distinctive in that it exploits the advantages of CuInSe_2_ QD sensitization and self-powered behavior while addressing the major limitations identified in previous studies, contributing to the continued development of high-performance, environmentally sustainable optoelectronic devices based on QDs—TiO_2_ heterostructures.

## 2. Materials and Methods

CuInSe_2_ QD_s_ were synthesized using the thermal injection method. The Se-TOP precursor was prepared by dissolving selenium powder in tri-n-octylphosphine (TOP), followed by sonication for 60 min. The resulting solution was then maintained at 120 °C. CuI and In(C_2_H_3_O_2_)_3_ were placed in a three-necked flask, followed by the addition of the reducing agent oleylamine (OLA) and 1-octadecene (ODE). Nitrogen was introduced to purge the system for 5 min at room temperature. The mixture was then heated to 170 °C and maintained at this temperature for 10 min. Subsequently, the Se-TOP precursor solution was injected into the flask over 3 min. The reaction was quenched by an ice-water bath, and methanol was added to perform three rounds of suspension precipitation. Finally, the precipitate was dispersed in toluene to obtain CuInSe_2_ QD_s_ [22].

An N-doped single-sided silicon oxide wafer was used as the substrate, on which a TiO_2_ electron transport layer was deposited via electron beam evaporation and annealed at 600 °C for 40 min. TiO_2_ photodetectors were fabricated by placing a mask plate on the TiO_2_ thin film layer, followed by sequential electron beam deposition of Au/Ti to preparate the electrodes. CuInSe_2_ QD_s_ were spin-coated onto the TiO_2_ photodetectors. A volume of 80 μL of the QDs solution was applied, and the substrates were spun at 2500 rpm for 30 s. This process was repeated five times with a 20 s interval between each deposition, and the devices were then dried for 15 min at 80 °C to complete the preparation.

The materials required for the experiment are listed below: 1-Octadecene(ODE, >90%), oleylamine(OLA, 80–90%), Tri-n-octylphosphine (TOP, 90%), Selenium (Se, 99.9%), Indium acetate (In(C_2_H_3_O_2_)_3_, 99.99%), cuprous iodide (CuI, 99.9%), methanol (CH_3_OH, 99.5%), n-hexane (C_6_H_14_, 97%) purchased from Shanghai McLean Biochemical Co., Ltd. (Shanghai, China). TiO_2_ Pellets, Ti Pellets, Au Pellets purchased from Zhongnuo New Material (Beijing) Technology Co., Ltd. (Beijing, China). Single-sided oxidized silicon wafers were purchased from Suzhou Crystal Silicon Electronic Technology Co., Ltd. (Suzhou, China).

## 3. Results and Discussions

**Figure 1:**
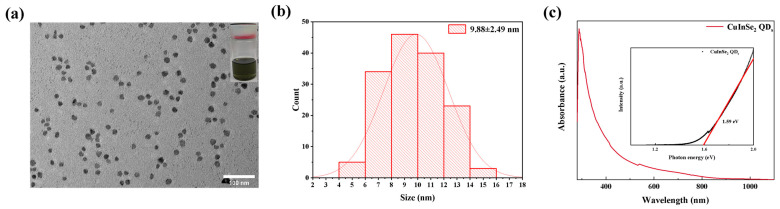
(**a**) TEM image of CuInSe_2_ QD_s_. (**b**) Particle size statistics of CuInSe_2_ QD_s_. (**c**) UV–visible absorption spectra of CuInSe_2_ QDs; the inset shows the bandgap diagram of CuInSe_2_ QDs.

The synthesized CuInSe_2_ QD_s_ were characterized, and Figure 1a,b present the TEM images and particle size distribution of the CuInSe_2_ QD_s_. The morphology of the QDs is an approximately circular shape, with an average size of ~9.88 nm, which is smaller than the 10.6 nm Bohr exciton radius of CuInSe_2_ QD_s_ [16]. Figure 1c shows the absorption spectrum of CuInSe_2_ QDs, with a range from 280 to 1100 nm; the strongest absorption peak occurs at ~300 nm, and the band gap of CuInSe_2_ QDs was calculated to be 1.59 eV according to the Tauc Plot method.

**Figure 2:**
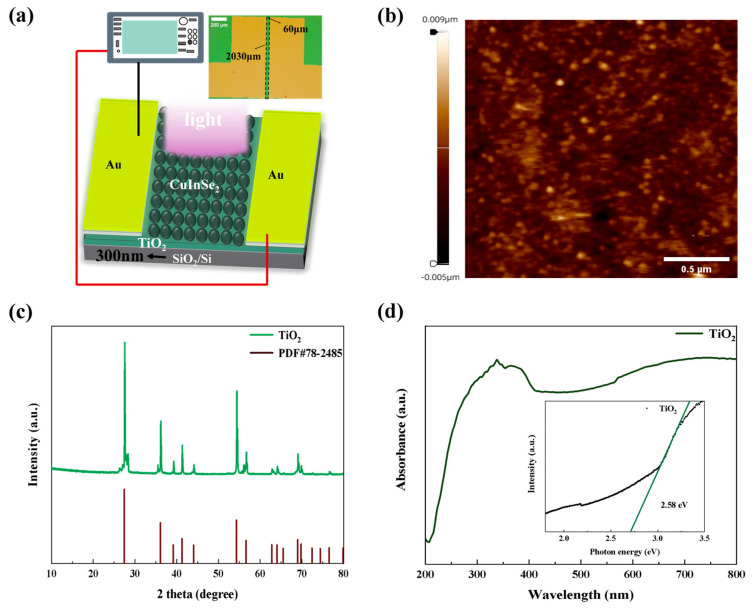
(**a**) Schematic diagram of CuInSe_2_ QD_s_ sensitization-enhanced TiO_2_ photodetector; the illustration shows the actual device. (**b**) Sensitized enhancement of TiO_2_ photodetector by CuInSe_2_ QD_s_ detector AFM diagram. (**c**) X-ray diffraction pattern of TiO_2_. (**d**) UV–visible absorption spectrum of TiO_2_; the inset shows the band gap diagram of TiO_2_.

Figure 2a depicts the cross-sectional architecture of the sensitized device, where a uniform monolayer of CuInSe_2_ QD_s_ is assembled on the mesoporous TiO_2_ electron-transport layer (ETL) through optimized spin-coating processing. In this configuration, the CuInSe_2_ QD layer functions as the primary photoactive component, achieving effective photon capture across visible to near-infrared spectra, while the underlying TiO_2_ scaffold serves dual functions: (i) facilitating rapid electron extraction from the QD_s_ through its tailored conduction band alignment and (ii) providing a continuous 3D charge transport network with minimized recombination losses. Figure 2b presents a representative AFM topography image of the sensitized device, revealing key morphological characteristics of the spin-coated CuInSe_2_ QDs/TiO_2_ heterostructure. Quantitative analysis shows that the root mean square roughness of the CuInSe_2_ QD-modified surface is 1.26 nm; the maximum height roughness is 14 nm, which is a 40% increase compared to the bare TiO_2_ substrate (10 nm). This controlled roughening demonstrates uniform QDs coverage while maintaining nanoscale ordering, and the spin-coated QDs mask some of the defective states, which makes the surface of the film denser. A moderate amount of roughness enhances the interfacial contact between TiO_2_ and CuInSe_2_ quantum dots, which promotes charge transfer, and the increase in surface roughness also leads to an increase in the effective area, which promotes better light absorption and reduces reflective losses, as well as generating localized surface defects, which serve as charge trapping centers and increase recombination [23]. Figure 2c shows the XRD patterns of the TiO_2_ layer. The figure shows several characteristic peaks which correspond to the Ref. [24], with the strongest peak at 2θ = 27.4°, corresponding to the 110 crystal plane, which exhibits a rutile phase [24]. Calculations based on Scherrer’s formula with the Williamson–Hall model [25] show that TiO_2_ films prepared by electron beam evaporation have a large grain size (59.76 nm), a low dislocation density (2.8 × 10^13^ m^−2^), and a small strain (0.018%), which is superior to that of typical sol-gel processes. Grain size optimization significantly reduces grain boundary scattering, and the combination of low defect density and lattice integrity synergistically improves carrier mobility and device stability. Figure 2d shows the absorption spectrum of TiO_2_ with the absorption peak at 350 nm, and the band gap of TiO_2_ was calculated to be 2.58 eV according to the Tauc Plot method, which directly affects its photoresponse in the visible wavelength band due to its forbidden band width [26,27]. This can also be verified from the photocurrents we tested on simple TiO_2_ photodetectors in the 565 nm band.

**Figure 3:**
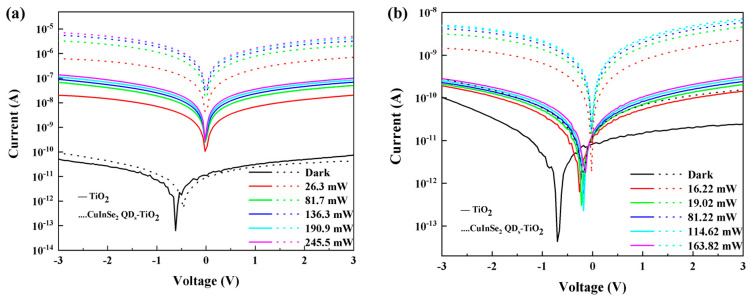
(**a**) TiO_2_ photodetector versus CuInSe_2_ QD-sensitized TiO_2_ photodetector IV curve at 405 nm. (**b**) TiO_2_ photodetector versus CuInSe_2_ QD-sensitized TiO_2_ photodetector IV curve at 565 nm.

Figure 3a,b show the IV output characteristics of TiO_2_ photodetectors versus TiO_2_ photodetectors enhanced by sensitization of CuInSe_2_ QD_s_ under the illumination of LEDs of different powers at 405 nm and 565 nm. The devices before and after sensitization show typical bipolar characteristics, especially the “V”-shaped transfer curves, which show the transfer of electrons or holes in the n-type or p-type channels of the devices, respectively, and the sensitized photocurrent exhibits a significant enhancement. For the self-powered behavior of the device, we calculated the open-circuit voltage *V*_oc_ and short-circuit current *I*_sc_ of the CuInSe_2_ QD-sensitized TiO_2_ photodetector at 405 nm illumination, which are 0.001 V and 4.62 × 10^−10^ A, respectively. The self-powered characteristics of the constructed device based on the heterostructure of CuInSe_2_ QDs/TiO_2_ were verified [28]. The switching ratio is the ratio of light current to dark current, as can be seen in Figure 3a; the sensitized device achieves a high switching ratio of about 105 at a 405 nm wavelength with a 3 V bias voltage of 245.5 mW, and the photocurrent increases with the increase in optical power. In Figure 3b, it can be seen that the photocurrent of the TiO_2_ photodetector at the 565 nm wavelength does not show significant growth with the increase in optical power, and the photocurrent shows a saturated state. The response of the device to light is significantly enhanced after sensitization with CuInSe_2_ QD_s_, allowing a detectable range up to 565 nm. In addition, the surface defects of CuInSe_2_ QD_s_ can quench any small current under dark conditions. Under light illumination, the photocarriers generated in CuInSe_2_ QD_s_ can rapidly fill the defects in the quantum dots, which can effectively collect the photocurrents in the thin films that are mainly generated by the TiO_2_ component [20].

**Figure 4:**
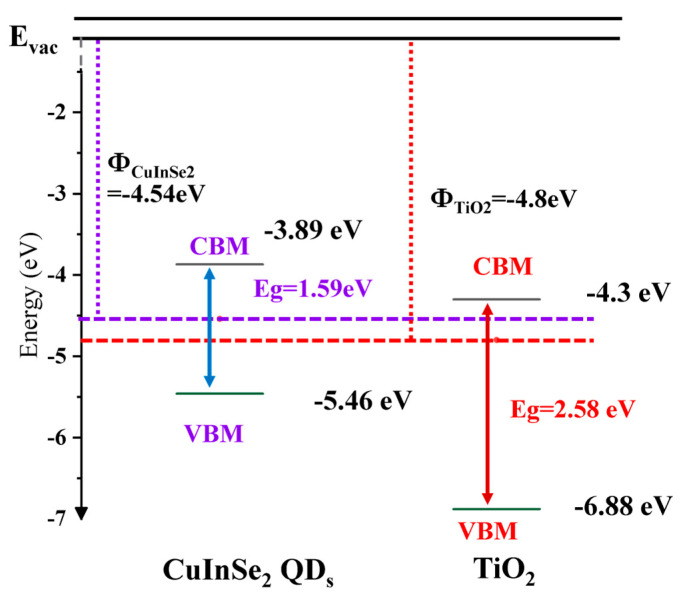
Energy band structure diagrams of CuInSe_2_ QDs and TiO_2_.

A type-II heterostructure between CuInSe_2_ QD_s_ and TiO_2_ is attributed to the improvement in photoresponsive properties [29]. The valence band offset between the CuInSe_2_ QDs and TiO_2_ is 1.42 eV. In addition, the conduction band offset calculated using the VBM and the bandgap of the two materials obtained by Tauc Plot fitting was determined to be about 0.41 eV [28]. According to the energy band theory, photogenerated electrons are spontaneously injected from the conduction band bottom of CuInSe_2_ QD_s_ into the conduction band bottom of TiO_2_, forming an energy level difference as a thermodynamic driving force, which causes the electrons to rapidly detach from the quantum dot interface and migrate to the external circuit. As shown in Figure 4, the photogenerated holes at the top of the TiO₂ valence band are transferred to the top of the valence band of CuInSe_2_ QD_s_ through the assistance of the interface’s built-in electric field, and the migration process is accelerated by the localized electric field formed by the bending of the energy bands inside the heterojunction [22,30]. This charge separation mechanism significantly reduces the compounding probability of electron-hole pairs and extends the carrier lifetime, thereby enhancing the photocurrent density. The energy band shift at the interface of the type-II heterojunction induces the formation of a built-in electric field (in the direction from CuInSe_2_ QD_s_ to TiO_2_), which further facilitates the injection efficiency of photogenerated electrons into the conduction band of TiO_2_ and at the same time drives the migration of holes into the valence band of the CuInSe_2_ QD_s_ to realize the fast spatial separation of carriers.

**Figure 5:**
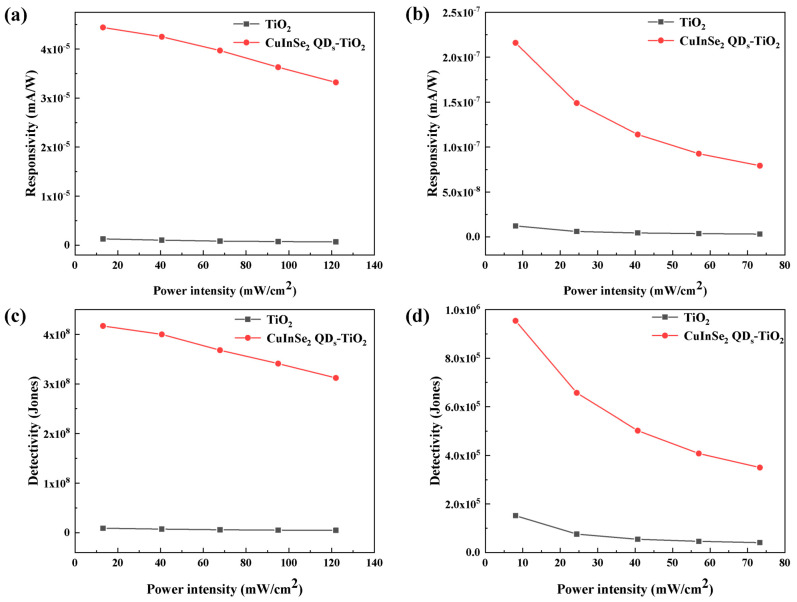
(**a**) Response curve of TiO_2_ photodetector sensitized with CuInSe_2_ QD_s_ at 405 nm. (**b**) Response curve of TiO_2_ photodetector sensitized with CuInSe_2_ QD_s_ at 565 nm. (**c**) Detectivity plot of TiO_2_ photodetector versus CuInSe_2_ QD-sensitized TiO_2_ photodetector at 405 nm. (**d**) Detectivity plot of TiO_2_ photodetector versus CuInSe_2_ QD-sensitized TiO_2_ photodetector at 565 nm.

The responsivity (*R*) is one of the key parameters of a photodetector, defined as the photocurrent generated per unit of effective incident power, and *R* can be calculated from Equation (1):

(1) $$R=\frac{\Delta I_{DS}}{P}=\frac{I_{illu}-I_{dark}}{E_{e}\times S}$$

where *I_illu_* is the generated photocurrent, *P* is the optical power density, *E*_e_ is the optical power density, and *S* is the effective area of the photosensitive region. The responsivity changes corresponding to the different optical powers of the two devices were calculated at a bias voltage of 3 V, as shown in Figure 5. The sensitized devices at both wavelengths show a significant increase in responsiveness compared to the original devices. Figure 5a shows the plot of *R* versus optical power at a wavelength of 405 nm. *R* reaches 4.44 × 10^−5^ mA/W at an optical power density of 13.1 mW/cm^2^. The sensitized responsivity is improved by 34 times over the 1.28 × 10^−6^ mA/W of the TiO_2_ device alone. Figure 5b represents the plot of *R* with optical power at a 565 nm wavelength, where the responsivity reaches 2.16 × 10^−7^ mA/W at an optical power density of 8.07 mW/cm^2^. Responsivity decreases with increasing light intensity; this dependence can be attributed to several factors, with a gradual decrease in responsivity due to enhanced carrier complexation and gradual saturation of the photocurrent [31,32]. At low light intensities, the trap state traps a large fraction of the photogenerated electrons, thus reducing the recombination of electron-hole pairs [33]. Conversely, at higher light intensities, heating effects and sensitized trap saturation in the quantum dots may also lead to reduced responsivity, including heterostructures and defects within their interfaces, and the availability of trap states that capture photogenerated electrons is relatively limited. As a result, the device is more responsive at lower light intensities [34].

Another key parameter characterizing the performance of the photodetector is the specific detectivity D*, which can be calculated by Equation (2) [35]:

(2) $$D^{*}=\frac{RA^{1/2}}{{\left(2eI_{ds}\right)}^{1/2}}$$

where *R* is the responsivity, *A* is the area of the detector, e is the charge of an electron, and *I*_ds_ is the dark current. The variation curve of D* with optical power at 2 V bias voltage is shown in Figure 5. Figure 5c represents the variation curve of detectivity with optical power density at 405 nm wavelength, and the detectivity of the sensitized device reaches 4.17 × 10^8^ Jones at a power density of 13.1 mW/cm^2^. Figure 5d represents the variation curve of detectivity with optical power density at a 565 nm wavelength; the D* at a 565 nm wavelength decreases with the increase in optical power density, showing the same behavior as the responsivity. The highest detection rate reaches 9.54 × 10^5^ Jones, which indicates that the device is more sensitive to the detection of weak light. The NEP is the least detectable power density for the dark noise by a PD with a 1 Hz bandwidth frequency and can be defined as [36].

(3) $$NEP=\frac{\sqrt{2e\times I_{ds}}}{R}$$

The NEP of the sensitized device at 3 V bias voltage (405 nm) was calculated and reached up to 9.49 × 10^−11^ W/Hz.

## 4. Conclusions

CuInSe_2_ QDs with an average size of 9.88 ± 2.49 nm were synthesized using the solution-phase synthesis method, and the TiO_2_-based photodetectors sensitized by the CuInSe_2_ QDs were prepared. The optimized device architecture synergized with QD-induced interfacial band engineering, achieving remarkable performance enhancement. The maximum switching ratio reached 10^5^ in the 405 nm band, and the responsivity reached 4.44 × 10^−5^ mA/W, a 34-times improvement over simple TiO_2_ devices. The detectivity reached 4.17 × 10^8^ Jones at 405 nm. The sensitized responsivity and detectivity exhibit the same photoresponse enhancement behavior, effectively allowing a detectable wavelength up to 565 nm. Thus, solution-based, low-cost processes and device performance enhancement make lead-free CuInSe_2_ QD_s_ promising materials for self-powered optoelectronic applications, and sensitization through quantum dots has become an effective method for photodetector performance enhancement.

## Data Availability

Data underlying the results presented in this paper are not publicly available at this time but may be obtained from the authors upon reasonable request.
